# Comparative investigation of the incidence of intraoperative seizures with propofol and remimazolam during anesthetic management for awake craniotomy: Retrospective propensity score matching study

**DOI:** 10.1097/MD.0000000000048946

**Published:** 2026-06-12

**Authors:** Yui Somura, Takehito Sato, Masashi Takakura, Kanako Ozeki, Takahiro Ando, Koichi Akiyama

**Affiliations:** a Department of Anesthesiology, Nagoya University Hospital, Nagoya, Aichi, Japan.

**Keywords:** awake craniotomy, propofol, remimazolam, seizure

## Abstract

Seizures are serious complications of awake craniotomy (AC), and generalized clonic seizures are problematic because they require pharmacologic termination and emergency airway management. Although associations between seizure onset and anesthetic agents such as propofol and dexmedetomidine have been reported, data on remimazolam are limited. This study aimed to investigate the characteristics of seizures in patients undergoing AC and to compare the frequency of intraoperative seizures associated with propofol and remimazolam administration for anesthetic management. The data of 328 patients who underwent AC at our hospital until September 2024 were retrospectively reviewed. They were divided into propofol (group P, n = 259) and remimazolam (group R, n = 69) groups. To ensure comparable baseline characteristics, propensity score matching was performed. After propensity score matching, 49 patients were selected from each group (Group R, n = 49; Group P, n = 49). Fifty-eight patients (17.6%) experienced seizures during AC. No significant difference in the frequency of seizures was observed between the groups anesthetized with propofol (Group P: 46 events) and remimazolam (Group R: 14 events; *P* = .37). Forty-nine patients were selected from each group after propensity score matching. The difference in the incidence of seizures was not significant (10 and 13 events in Groups P and R, respectively; *P* = 1.00). Binary logistic regression analysis revealed no association between seizure occurrence and anesthetic management with remimazolam (*P* = .18). However, younger patients were more likely to experience seizures during AC (odds ratio, 0.95; 95% confidence interval, 0.935–0.983; *P* < .01). The similar frequencies of seizures associated with the use of remimazolam and propofol suggest that remimazolam might not increase the risk of intraoperative seizures during AC. These findings indicate that remimazolam may be safely used as an anesthetic for AC.

## 1. Introduction

Seizures are a severe complication of awake craniotomy (AC), and the occurrence of clonic seizures is especially problematic because they necessitate the administration of medications to abort the seizure and emergency airway management.^[1,2]^ Remimazolam, an ultra-short-acting intravenous benzodiazepine anesthetic, was introduced in Japan in 2020,^[3]^ and has been used to provide anesthesia during AC.^[3–5]^

The incidence of seizures associated with the use of anesthetic drugs such as propofol and dexmedetomidine has been compared and reported.^[6]^ However, such investigations for remimazolam are lacking. If the use of the benzodiazepine anesthetic remimazolam does not change the frequency of seizures, it may be possible to use it in awake surgery in a manner equivalent to that of conventional drugs, such as propofol.

Furthermore, the use of the antagonist flumazenil may induce seizures; however, few reports have been published on this, and no reports or evidence exist concerning AC.

Therefore, we investigated the characteristics of seizures in patients undergoing AC and the frequency of intraoperative seizures associated with propofol and remimazolam administration for anesthetic management.

## 2. Materials and methods

This retrospective study was approved by the Ethics Committee of Nagoya University (approval number: 2024-0314). Informed consent was obtained from all participants using the opt-out method. This study is reported in accordance with the Strengthening the Reporting of Observational Studies in Epidemiology reporting guidelines.

The inclusion criteria were as follows: brain tumor in the frontal or temporal lobe requiring intraoperative language tasks or focal resection for epilepsy near the language areas, necessitating the patient to be awake during the resection; and age of 18 to 70 years.

The exclusion criteria were as follows: American Society of Anesthesiologists physical status score > 3; liver failure (Child–Pugh classification B or C); severe respiratory disease (such as severe obstructive pulmonary disease) and respiratory dysfunction (vital capacity < 60% or forced expiratory volume in 1 second < 50%); and hemostatic dysfunction (platelet count < 80,000/μL, prothrombin time-international normalized ratio > 1.5, or activated partial thromboplastin time > 40 seconds).

### 2.1. Anesthetic management

All patients underwent AC following the asleep-awake-asleep protocol. General anesthesia was induced via continuous infusion of remimazolam besylate (Anerem 50 mg; Mundipharma K.K.) at 12 mg/kg/h in the remimazolam group (Group R). The dose was reduced after loss of consciousness. Propofol (1% Diprivan Injection kit; Aspenpharma) was administered to the propofol group (Group P) via target-controlled infusion at a concentration of 3 μg/mL. Fentanyl (25–100 μg) and remifentanil (0.1–0.2 µg/kg/min) were also used for anesthesia induction in both groups. Anesthesia was maintained with remimazolam (0.5–1.0 mg/kg/h) or propofol (2.5–4.5 μg/mL via target-controlled infusion) in conjunction with remifentanil (0.1–0.25 µg/kg/min) to maintain their bispectral index monitor (Philips) values between 40 and 60.

Supraglottic airways (SGAs) (I-gel or Proseal) were inserted for airway management. All patients underwent general anesthesia and scalp blocks. Ropivacaine (0.375%) was used for scalp blocks and at the headpin site, surgical site, temporal muscle, and dura. Bilateral scalp blocks (supraorbital, supratrochlear, greater occipital, lesser occipital, auriculotemporal, and zygomaticotemporal nerves) were administered to minimize intraoperative pain.

Small doses of rocuronium (10–40 mg) were administered during the induction and intraoperative maintenance of anesthesia, as required. Analgesia was adjusted to maintain systolic blood pressure at < 140 mm Hg during the sleep phase of the procedure. Oxygenation was maintained at > 94%, and the heart rate was maintained at 50–100 beats/min.

Anticonvulsants (levetiracetam [500 mg] and fosphenytoin [750 mg]) were administered before the awakening phase to prevent intraoperative seizures. Mannitol (150–300 mL) was administered to reduce intracranial pressure. Low-dose dexamethasone (6.6 mg) or granisetron (3 mg) was administered as an antiemetic agent at the discretion of the anesthesiologist.

Anesthetic agent infusion was stopped when the neurosurgeon visualized the brain tumor lesion. The SGA was removed after the patients regained consciousness and were able to respond to verbal communication, which marked the start of the awake phase. The patients in the R group received flumazenil as needed after the recovery of spontaneous breathing.

Metoclopramide (10 mg) was administered to treat nausea. Patients received local anesthetic (1% lidocaine with epinephrine 1:100,000), flurbiprofen (50 mg), acetaminophen (1000 mg or 15 mg/kg if weight was < 50 kg), and low-dose fentanyl (25 μg) for analgesia. Intraoperative hypertension was defined as elevated blood pressure (> 20% of the baseline blood pressure or systolic blood pressure of > 150 mm Hg) during the awake phase.

We recorded partial seizures (facial or limb seizures) and clonic convulsions. We also recorded the electroencephalography findings after discharge. If a seizure occurred, the neurosurgeon administered cold water to the brain surface in the surgical field. The anesthesiologist administered anticonvulsants and induced general anesthesia if necessary.

The patients underwent intraoperative magnetic resonance imaging after tumor resection, followed by the induction of general anesthesia. The SGA was then reinserted via the lateral caudal approach. The patients were awakened from anesthesia and transferred to the intensive care unit after surgery.

### 2.2. Outcome measure and statistics

The primary outcome measure was the number of seizures. Specifically, we determined whether the number of seizure events increased with remimazolam relative to propofol during the emergent phase of AC. We also investigated the factors associated with the onset of seizures during the awake phase. The secondary outcomes were the frequencies of other intraoperative complications, such as pain, hypertension, and vomiting, as well as postoperative nausea and vomiting.

Factors that may influence intraoperative seizures during AC were matched using propensity score matching (PSM). The 2 groups were matched in a 1:1 ratio based on 6 covariates (age, sex, body mass index, awake time, dose of fentanyl before the awake phase, and anticonvulsants). The nearest-neighbor matching method (1:1 ratio) was applied with a caliper width of 0.2 for the logit-transformed propensity score. The variables in the matched dataset were considered balanced between the groups if the standardized mean difference was < 0.1. The *C*-statistic of the propensity score model before matching was 0.87, indicating adequate discrimination.

Categorical variables are expressed as numbers (percentages), and continuous variables are expressed as medians (interquartile ranges). Data were analyzed using Fisher exact test and Mann–Whitney *U* test. Logistic regression analysis was performed to identify the factors influencing intraoperative seizures.

Differences were considered statistically significant at *P* < .05. Statistical analyses were performed using EZR and R version 2.9.1 (The R Foundation for Statistical Computing).

## 3. Results

We retrospectively investigated 328 cases of AC performed at our hospital between January 1, 2006, and September 30, 2024. The participants were divided into the propofol (Group P; n = 259) and remimazolam (Group R; n = 69) groups. After PSM, 49 patients were selected from each group (Group R, n = 49; Group P, n = 49).

Before PSM, significant differences were observed in patient characteristics, particularly awake time, amount of fentanyl used until awakening, amount of intraoperative blood loss, and amount of fluid transfusion between the R and P groups; however, these differences disappeared after matching (Table 1).

**Table 1:** Baseline characteristics and intraoperative data of the 2 groups before and after PSM.

	Before propensity matching			After propensity matching		
	Group P (n = 259)	Group R (n = 69)	*P* value	Group P (n = 49)	Group R (n = 49)	*P* value
Age (Yrs)	42.00 (33.5–50.75)	44.00 (37.00–51.00)	.23	43.00 (32.00–51.00)	44.00 (37.00–49.00)	.52
Sex (male/female)	105/154	24/45	.4	19/30	16/33	.67
Height (cm)	167.00 (159.50–172.00)	167.80 (159.0–172.0)	.86	167.00 (159.00–174.00)	168.00 (160.00–172.00)	.73
Weight (kg)	61.70 (54.05–70.22)	62.80 (54.50–75.00)	.51	63.70 (55.00–71.40)	61.50 (54.50–72.40)	.83
BMI (kg/m^2^)	22.30 (20.13–24.55)	22.30 (20.57–25.68)	.35	22.80 (20.16–24.75)	21.50 (20.02–25.00)	.80
Awake time (min)	139.0 (104.0–180.0)	124.0 (100.0–146.0)	.006	119.0 (94.00–148.00)	12,700 (102.00–146.00)	.46
Anesthesia time (min)	561.0 (506.0–657.0)	539.0 (502.0–619.0)	.25	586.0 (519.0–677.0)	536.0 (512.0–624.0)	.17
Bleeding (mL)	392.0 (216.25–672.00)	323.0 (154.00–480.00)	.01	346.0 (156.0–610.0)	306.0 (175.00–428.0)	.30
Infusion (mL)	3630.0 (3050.0,4287.0)	4200.0 (3600.0–4800.0)	< .01	4100.0 (3450.0–4870.0)	4060.0 (3540.0–4800.0)	.84
Fentanyl before awake phase (μg)	200.00 (100.00–250.00)	50.00 (25.00–100.00)	< .01	75.00 (50.00–100.00)	75.00 (50.00–150.00)	.23
Highest systolic arterial blood pressure (mm Hg)	148.00 (131.00–161.00)	146.00 (128.00–159.00)	.27	150.0 (126.0–161.0)	145.00 (127.00–158.00)	.83
Time needed to awake (min)	17.00 (13.75–21.25)	17.00 (14.00–20.00)	.78	16.00 (13.25–23.00)	17.00 (14.00–20.00)	.64
Flumazenil (%)	0	60 (86.0)		0	41 (83.7)	
Hypertension (%)	142 (55.0)	35 (50.7)	.58	19 (46.3)	18 (43.9)	1.00
Vomiting (%)	15 (5.8)	9 (13.0)	.06	2 (4.1)	7 (14.3)	.15
Pain (%)	106 (41.1)	33 (47.8)	.33	26 (53.1)	25 (51.0)	1.0
Agitation (%)	19 (7.4)	3 (4.3)	.58	2 (4.1)	3 (6.1)	1.0

Values are presented as median (IQR).

Group P = propofol group, Group R = remimazolam group, IQR = interquartile range, n = number of patients, PSM = propensity score matching.

Statistical significance was set at *P* < .05.

Fifty-eight patients (17.6%) experienced seizures during AC (Table 1). These were focal seizures involving the face and upper and lower limbs. The seizures were terminated by the administration of cold water to the cerebral surface in the surgical field. Most seizures occurred during stimulation for language mapping. The frequency of intraoperative seizures did not differ significantly between the groups (46 events in Group P vs 14 events in Group R; *P* = .37). The types of seizures did not differ significantly (Table 2).

**Table 2:** Seizure events.

	Before propensity matching			After propensity matching		
	Group P (n = 259)	Group R (n = 69)	*P* value	Group P (n = 49)	Group R (n = 49)	*P* value
Seizure (%)	46 (17.8)	14 (20.3)	.60	9 (18.4)	9 (18.4)	1.0
Facial seizure (%)	18 (7.0)	7 (10.1)	.44	1 (2.0)	5 (10.2)	.2
Limb seizure (%)	22 (8.5)	8 (11.6)	.48	7 (14.3)	4 (8.2)	.52
Clonic convulsion (%)	4 (1.6)	1 (1.4)	0	1 (2.0)	1 (2.0)	1.0
After discharge (%)	45 (17.4)	7 (10.1)	.19	5 (10.2)	6 (12.2)	1.0
Anticonvulsant: levetiracetam use (%)	132 (51.2)	63 (91.3)	< .01	41 (83.7)	43 (87.8)	.77

Values are presented as median (IQR).

Group P = propofol group, Group R = remimazolam group, IQR = interquartile range, n = number of patients.

Statistical significance was set at *P* < .05.

No significant differences were observed in the number (Group P: 10 events vs Group R: 13 events; *P* = 1.00) or types (Table 2) of seizures after PSM.

Fifty-five patients in the remimazolam group received flumazenil before PSM, and none experienced seizures after its administration.

Two patients (0.6%) in the Group P had generalized clonic convulsions that were not resolved with cold water administration to the surgical field. They required emergency intubation and induction of general anesthesia (low-dose propofol administration).

Logistic regression analysis also revealed no association between the incidence of seizures and anesthetic management using remimazolam (odds ratio, 1.730; 95% confidence interval, 0.809–3.690; *P* = .18). Younger age was associated with the onset of seizures during AC (odds ratio, 0.95; 95% confidence interval, 0.935–0.983; *P* < .01) (Table 3).

**Table 3:** Logistic regression analysis data.

	OR	95% CI	*P* value
Age	0.950	0.930–0.983	< .01
Remimazolam	1.730	0.809–3.690	.18

CI = confidence interval, OR = odds ratio.

## 4. Discussion

Our retrospective study showed that the incidence of seizures in AC was similar between remimazolam and propofol, and no overt seizures were observed with flumazenil.

Intraoperative seizures are a common complication of AC.^[1,2,7]^ These seizures cause discomfort and reduce alertness, which hinders task performance. They require careful consideration during anesthetic management, as they can lead to AC failure and prolong hospital stay.^[2]^

The reported incidence of seizures during AC is 3 to 30%.^[1,2,6,7]^ The risk factors for intraoperative seizures include high stimulation potential and frequent electrical stimulation during mapping,^[2,6]^ frontal lobe tumors,^[7]^ and a history of preoperative anticonvulsant medication.^[7,8]^ Anticonvulsants administered before the awakening phase of AC have been reported to be effective preventive measures.^[2,9]^

Propofol has anticonvulsant properties, which are a significant advantage in AC management. Dexmedetomidine is also widely used for AC.^[10]^ However, a previous study reported that its administration is a risk factor for seizures during AC.^[6]^ The frequency of seizures was higher in the dexmedetomidine group than in the Group P in the study, but logistic regression analysis revealed no statistically significant increase in the risk of seizures.^[6]^

No reports have been published on intraoperative seizures during AC with remimazolam. Remimazolam is an ultra-short-acting benzodiazepine with an efficacy comparable to that of propofol for inducing and maintaining general anesthesia.^[11]^

Previous reports have not found a significant difference in the incidence of seizures associated with remimazolam anesthesia during AC^[3,5]^; however, this was a secondary endpoint. The primary endpoint of this study was seizures; no significant differences were observed in their incidence.

The effects of remimazolam can be antagonized by flumazenil. Remimazolam has the advantage of enabling clear wakefulness during AC.^[5]^ Previous studies have reported that anesthesia using a combination of remimazolam and flumazenil does not increase the risk of perioperative seizures compared with total intravenous anesthesia using propofol.^[12]^ However, convulsions and restlessness have been reported after the administration of flumazenil during anesthesia management with benzodiazepine anesthetics.^[13]^

The risk is particularly high in patients undergoing reversal from deep sedation and in those receiving benzodiazepines for a long period.^[13]^

In such cases, flumazenil should be avoided. However, it should be administered with caution if necessary. None of the patients in this study had been taking benzodiazepines for a long period, and flumazenil was administered when signs of awakening were observed.^[5]^ Therefore, safe reversal of the anesthetic effect is considered possible.

Younger patients reportedly experience intraoperative seizures more frequently during AC. A single-center retrospective study reported that intraoperative seizures during AC tended to be more common in younger patients.^[14]^ However, systematic reviews and multivariate analyses have not consistently demonstrated young age to be an independent risk factor.^[7]^

Young age is presumably a related factor reflecting background factors such as tumor location,^[14]^ cortical excitability,^[15,16]^ and coexisting epilepsy^[17]^; however, no established evidence has been found.

One plausible explanation is the higher cortical excitability and plasticity observed in younger brains. Neurophysiological studies using paired associative stimulation and transcranial magnetic stimulation have demonstrated that cortical plasticity and excitability decline with aging, indicating that younger cortices respond more strongly to external stimulation such as intraoperative electrical mapping.^[15,16]^

Furthermore, younger patients more frequently harbor epilepsy-associated tumors, including low-grade gliomas and long-term epilepsy-associated tumors, which are intrinsically epileptogenic and present with seizures in the majority of cases.^[17,18]^ In addition, developing and young adult brains may exhibit higher network plasticity and synchronization, potentially lowering the seizure threshold in response to cortical stimulation.^[15–17,19]^

Taken together, younger age may not represent an independent risk factor per se, but rather reflects a neurobiological milieu characterized by heightened cortical responsiveness and a higher prevalence of epilepsy-prone tumor pathology.

This study suggests that the use of remimazolam in young patients may increase the risk of intraoperative seizures during AC, and that these factors may be involved. In any case, careful attention should be paid to the occurrence of intraoperative seizures in young patients.

In addition, previous reports have shown that remimazolam results in faster and better recovery than propofol in AC.^[5]^ The results of this study showed that remimazolam does not necessarily increase the frequency of convulsions, suggesting that it can be used satisfactorily for anesthesia in AC.

This study has some limitations. These include its retrospective design and the lack of standardization of the types of anticonvulsants used during AC. We performed PSM to mitigate these limitations. Furthermore, data on the stimulation current strength during intraoperative mapping were not standardized and could not be obtained. The likelihood of seizures increases with stimulation intensity and the number of consecutive stimuli during awake mapping.^[20,21]^ Therefore, a prospective randomized trial that meets these criteria is required.

## 5. Conclusion

In conclusion, the incidence of seizures during AC was similar for remimazolam and propofol. No obvious seizures attributable to flumazenil administration were observed. These findings suggest that remimazolam does not significantly affect the occurrence of seizures during AC and support its use for anesthetic management in AC. Thus, remimazolam may be safely used as an anesthetic for AC.

## Acknowledgments

We would like to thank Editage (www.editage.jp) for English language editing.

## Author contributions

**Investigation:** Yui Somura, Takehito Sato.

**Methodology:** Yui Somura, Takehito Sato.

**Supervision:** Kanako Ozeki, Takahiro Ando, Koichi Akiyama.

**Writing – original draft:** Yui Somura.

**Writing – review & editing:** Takehito Sato, Masashi Takakura, Kanako Ozeki, Takahiro Ando, Koichi Akiyama,
